# Mitigating losses: how scientific organisations can help address the impact of the COVID-19 pandemic on early-career researchers

**DOI:** 10.1057/s41599-021-00944-1

**Published:** 2021-11-19

**Authors:** Sandra López-Vergès, Bernardo Urbani, David Fernández Rivas, Sandeep Kaur-Ghumaan, Anna K. Coussens, Felix Moronta-Barrios, Suraj Bhattarai, Leila Niamir, Velia Siciliano, Andreea Molnar, Amanda Weltman, Meghnath Dhimal, Shalini S. Arya, Karen J. Cloete, Almas Taj Awan, Stefan Kohler, Chandra Shekhar Sharma, Clarissa Rios Rojas, Yoko Shimpuku, John Ganle, Maryam M. Matin, Justine G. Nzweundji, Abdeslam Badre, Paulina Carmona-Mora

**Affiliations:** 1 Gorgas Memorial Institute of Health Studies, Sistema Nacional de Investigación SNI del SENACYT, Panama City, Panama; 2 Center for Anthropology, Venezuelan Institute for Scientific Research, Caracas, Venezuela; 3 University of Twente, Enschede, The Netherlands; 4 Young Academy of Europe, Cardiff, UK; 5 University of Delhi, New Delhi, India; 6 Organization for Women in Science for the Developing World, Trieste, Italy; 7 Walter and Eliza Hall Institute of Medical Research, Parkville, VIC Australia; 8 University of Cape Town, Cape Town, South Africa; 9 International Centre for Genetic Engineering and Biotechnology, Trieste, Italy; 10 Global Institute for Interdisciplinary Studies, Lalitpur, Nepal; 11 National Young Academy of Nepal, Kathmandu, Nepal; 12 International Institute for Applied Systems Analysis (IIASA), Laxenburg, Austria; 13 Mercator Research Institute on Global Commons and Climate Change, Berlin, Germany; 14 Istituto Italiano di Tecnologia, Naples, Italy; 15 Swinburne University of Technology, Hawthorn, VIC Australia; 16 Early- and Mid-Career Researcher Forum, Melbourne, VIC Australia; 17 Nepal Health Research Council, Kathmandu, Nepal; 18 Institute of Chemical Technology, Mumbai, India; 19 Indian National Young Academy of Sciences, New Delhi, India; 20 UNESCO-UNISA Africa Chair in Nanosciences/Nanotechnology, College of Graduate Studies, University of South Africa, Pretoria, South Africa; 21 Nanosciences African Network (NANOAFNET), iThemba LABS-National Research Foundation, Somerset West, South Africa; 22 University of Sao Paulo, Sao Paulo, Brazil; 23 National Young Academy of Young Scientists Pakistan, Lahore, Pakistan; 24 Heidelberg University, Heidelberg, Germany; 25 Indian Institute of Technology, Hyderabad, India; 26 Centre for the Study of Existential Risk, University of Cambridge, Cambridge, UK; 27 Hiroshima University, Hiroshima, Japan; 28 Young Academy of Japan, Tokyo, Japan; 29 University of Ghana, Accra, Ghana; 30 Department of Biology, Ferdowsi University of Mashhad, Mashhad, Iran; 31 Institute of Medical Research and Medicinal Plants Studies, Yaounde, Cameroon; 32 Cameroon Academy of Young Scientists, Yaounde, Cameroon; 33 Mohammed V University in Rabat, Rabat, Morocco; 34 Department of Neurology and MIND Institute, University of California-Davis, Sacramento, CA USA

**Keywords:** Science, technology and society, Health humanities

## Abstract

Scientific collaborations among nations to address common problems and to build international partnerships as part of science diplomacy is a well-established notion. The international flow of people and ideas has played an important role in the advancement of the ‘Sciences’ and the current pandemic scenario has drawn attention towards the genuine need for a stronger role of science diplomacy, science advice and science communication. In dealing with the COVID-19 pandemic, visible interactions across science, policy, science communication to the public and diplomacy worldwide have promptly emerged. These interactions have benefited primarily the disciplines of knowledge that are directly informing the pandemic response, while other scientific fields have been relegated. The effects of the COVID-19 pandemic on scientists of all disciplines and from all world regions are discussed here, with a focus on early-career researchers (ECRs), as a vulnerable population in the research system. Young academies and ECR-driven organisations could suggest ECR-powered solutions and actions that could have the potential to mitigate these effects on ECRs working on disciplines not related to the pandemic response. In relation with governments and other scientific organisations, they can have an impact on strengthening and creating fairer scientific systems for ECRs at the national, regional, and global level.

## Introduction

Despite a lack of early coordinated responses at national and multinational levels (Colglazier, 2020), the global emergence of the Coronavirus Disease 2019 (COVID-19) pandemic promoted unprecedented cooperative actions on the science-policy, science-communication, and science-diplomacy interfaces. With varying degrees of success, various actions within the realm of science diplomacy have accompanied the creation of knowledge related to COVID-19 through international collaboration (*Access to COVID-19 Tools (ACT) Accelerator* (2021), *COVID-19 Vaccine Tracker and Landscape*, (2021); Hadfield et al., 2018; Zhou et al., 2020)^1^. The COVID-19 pandemic brought the world to a standstill and also drew attention to the need for science diplomacy (Gore et al., 2020; *Here’s How “science Diplomacy” Can Help Us Contain COVID-19* (2021), New frontiers in science diplomacy, 2010; Pisupati, 2019), which refers to the use of scientific collaborations among nations to address common problems and to build international partnerships. This pandemic highlighted the need for researchers to be prepared to interact at the interface of diplomacy and policy, as well as science communication. Indeed, scientists need to be able to communicate their scientific knowledge (and the uncertainty attached to it) on public health issues to policymakers and to general audiences, helping the understanding of the advice and guidelines provided by international organisations of experts (i.e., the World Health Organisation, WHO). Then, the continuously changing scientific information can be better understood and accepted, and thus, used for measures during and after the pandemic. The participation of regional or national scientists, including early (and middle) career researchers (ECRs), during decisions, and in science communication activities with the public could increase the understanding and trust on science and on measures based on scientific evidence. The general population reacts better to scientists with similar language and culture, that they could feel related to. However, to do this, it is necessary to create or strengthen specific platforms for experts to interact with decision-makers at the national and international levels, especially in the Global South. While there are some platforms for such nexus, the Global South needs representation of their own scientists in the expert panels, as well as representation of disciplines that were not as involved in health advice before the pandemic.

The pandemic inevitably shifted the focus on scientific areas that directly address the pandemic response, namely epidemiology, public health, virology, immunology, pharmacology, human behavioural research, economics, anthropology, sociology, political science, among others. At the same time, some scientific areas not related to the pandemic response have been slowed down—due to inevitable physical and social restrictions^2^ that impacted certain aspects of academic life. This does not only relate to daily access to laboratories, scientific collections, or field sites, in person attendance to international meetings and events, exchange programs strengthening international collaborations, but also associates with furloughed employees and reduced funding to non-COVID-19-related research (Boosting Research without Supporting Universities Is Wrong-Headed, 2020; Corlett et al., 2020; Kent et al., 2020; Subramanya et al., 2020). Moreover, during the pandemic, scientists with children, especially women and even more early-career women researchers, have had to reduce their research efforts substantially, with future implications still difficult to quantify (Bittante et al., 2020; Myers et al., 2020). While some researchers might have focused during telecommuting and lockdown periods on data analysis and publication writing, there is evidence that the existing gender gap in academia (Oleschuk, 2020; Viglione, 2020) has increased with the pandemic. This is expected to have long-term consequences on reaching gender equity in all scientific fields and academic levels (Viglione, 2020)^3^. This increase in gender disparity in science has been even higher in some countries, and thus will have a more negative impact in their national science systems. These existing challenges in research are aggravated by the pandemic and cause long-term effects due to reduced capacity for generating pre-requisite data for securing new funding, termination of research, reduced networking opportunities, restrictions to international collaborations, collectively having a negative impact on other disciplines. Early-career researchers (ECRs) represent a highly vulnerable population and could be more affected by these issues (Korbel and Stegle, 2020). ECRs refer to Ph.D students, postdoctoral fellows and scientists who have 10 years or less of experience after the doctoral degree, although there is some flexibility in this definition (Bazeley, 2003). Owing to their career stage, ECRs often face job precarity, lack of available opportunities, low funding, and job insecurity (e.g., untenured positions and temporary contract employees) (Oleschuk, 2020). These problems are augmented by the COVID-19 pandemic and may be experienced more in science-lagging countries. These countries’ research environment depends on training their ECRs and conducting experiments abroad through international exchanges programs^4^, which have been stopped due to the pandemic.

Given these unprecedented times in global science, many international scientific organisations, policymakers, scientific communities, and private stakeholders have strengthened their collaborations in response to the pandemic (Ziegler, 2020). We, as ECRs, strongly think that science diplomacy can make a difference in addressing the current and future challenges at the global and regional level that ECRs would face inside and outside academia (emanated or amplified by the current pandemic). Here, we present such challenges, and discuss how national, regional and global organisations of early to mid-career researchers, like the Global Young Academy (GYA), National Young Academies, and others, could provide a platform for ECRs to train or practice science diplomacy, science advice, and science communication that would then allow them to give a voice to ECRs themselves to suggest possible solutions to their own challenges and to participate in the strengthening of the scientific systems of their countries. The former by serving as a bridge between ECRs and governments, the second by hearing the voices of young researchers when enacting policies, and the latter by informing societies about the benefits of scientific research. We describe and suggest measures for scientists, funding agencies, and international organisations that could help foster international collaboration.

## Effects of the COVID-19 pandemic on science and early-career researchers

The COVID-19 pandemic has affected science globally at multiple levels and scales, including academia, transnational stakeholders, governmental agencies, non-state actors, and industries. Scientific fields directly related to the pandemic response (fields mentioned in the introduction), received special attention at the national, regional, and global levels in terms of funding priorities, continuity of in-person activities during movement restrictions, as well as public awareness about their relevance to the society. The urgency of the pandemic response prompted the creation of international collaborations (e.g., diagnosis, vaccines and therapeutics development, viral genome sequencing, and clinical trials) often supported with special, rapid turnaround funding opportunities.

Another collateral but unexpected effect of the pandemic are enhanced virtual interactions observed across disciplines. Conferences, workshops, and seminars that moved to digital formats often became more inclusive and enabled scientists to potentially reach broader audiences. International academic mobility and scientific exchange are commonly threatened by limited travel budget and visa-related issues (Nshemereirwe, 2018), then inadvertently, virtual meetings addressed inclusiveness concerns, especially for ECRs from countries with strong mobility issues. Nevertheless, the lack of in-person interactions may not allow sufficient networking opportunities for the initiation of collaborations, or career opportunities for ECRs, especially outside their countries, often a scientific training step for ECRs from low- and middle-income countries (Fleming, 2020; Porpiglia et al., 2020; Termini and Traver, 2020). The positive and negative effects of virtual engagement have been discussed, including inequities in technology access and personal issues associated with telecommuting, such as being primary caregivers of children—which has widened gender disparities for women scientists (Gewin, 2020; Muric et al., 2020).

Quarantines in many countries, concomitant travel restrictions, closures of laboratories, delays in procuring laboratory equipment and supplies, stalled progress in research other than COVID-19-related, are all reasons for many scientists to not attend conferences planned before the pandemic was declared, reducing the chances for engaging with other researchers. Additionally, researchers need to secure funding, which usually depends on the progress of ongoing research. The issues inherent to the pandemic contingency may not provide the conditions to advance research for securing future funding. This has a higher effect on ECRs whose salaries generally depend directly on their grants or fellowships (Bégin-Caouette et al., 2020; “Introductions to the Community: Early-Career Researchers in the Time of COVID-19,” 2020). This scenario could improve if researchers create new partnerships, especially between countries with different management of the COVID-19 contingency, or that are at different stages of the pandemic. Thus, research tasks could be shared and complemented if performed in different locations or sharing facilities where the contingency allows in-person work, as part of a collaboration with researchers where research is stalled, allowing for continuity.

The issues affecting ECRs working in disciplines not directly related to the pandemic response also worsened the plight of some subgroups, particularly postdoctoral researchers and those finishing their Ph.Ds, impacting career plans, expiration of fellowships, and wellbeing (Paula, 2020; Postdocs in Crisis: Science Cannot Risk Losing the next Generation, 2020)^5^,^6^. Owing to the reduction in research funding, there could be an effect on employment and contract-research workers, a common scenario for ECRs, making them prone to job insecurity. The insecurities of scientific careers may discourage postdoctoral researchers and up-coming generations of science graduates from adopting scientific research as a lifelong career or even dropping scientific careers both in the academic and private sectors. This may disproportionately affect women, underrepresented groups, and science-lagging countries (Myers et al., 2020; We”re Losing an Entire Generation of Scientists.’ COVID-19’s Economic Toll Hits Latin America Hard, 2020). Furthermore, these issues would also impact higher education as ECRs have a relevant role in teaching/education, especially at the undergraduate level and the formation of new researchers. Science diplomacy and international organisations could help design international exchange and engagement programs to support ECRs continue their research in the current and post-pandemic times (specific suggestions can be found in Table 1).

**Table 1:** Recommendations to create and/or maintain a scientific environment that can foster new international opportunities for ECRs to allow research continuity during and post-pandemic.

Challenge	Recommendation	Goal	Measures of success	Science advice and diplomacy component
Reduced visibility and networking opportunities for ECRs	For young researchers: Connect digitally with other researchers, regionally and globally. Membership to a relevant organisation to facilitate asynchronous interactions with a wide array of colleagues	New opportunities for partnerships to increase collaborations and competitiveness, fostering technical exchange and scientific excellence	Research output (publications, briefings, etc.) metrics, funding adjudication, broader impact of research	Promotion of intergovernmental associations for fostering mobility of researchers between countries
	For international societies, academies, and organisations: Establish/enhance mentoring programs (at the intergenerational and international levels)	Facilitate the transfer of experience and create bridges of mentoring and collaborations	Acquisition of leadership skills, case by case on novel professional development opportunities and new collaborations	Furthering the circulation of scientific experiences at multinational levels, with focus in less scientifically developed countries
	International societies, academies, and organisations: create capacity building opportunities to empower leadership	Access to a highly skilled scientific workforce to work together in the interphases of policy and diplomacy with regional or global perspective	Increased visibility and representation of ECRs in science advice and diplomacy	Facilitate tailored programs for creating coherent international policies to avoid brain drain and loss, and stimulate innovation by young researchers
Reduction of international collaborations, especially for science-lagging or ODA-recipient countries	Funding agencies: enhance or create funding that allows the creation of new collaborative projects, including pilots and exploratory studies opened to applicants from all professional stages. Encourage the inclusion of science-lagging countries in international consortiums. Flexibility in timelines and extension supplements for active projects	Connect countries through science by creating global fellowships for ECRs to establish new collaborations and continue their research, or gather preliminary data to secure future funding	Research output (publications, briefings, etc.) metrics, funding acquisition, successful continuity of ongoing projects. broader impact of research	Implement collaborative scientific schemes with national, regional, and transnational stakeholders to promote scientific exchange
Augmented impact on science-lagging or ODA-recipient countries’ ECRs	International societies, academies, and organisations: organise activities that foster a collaborative and inclusive environment for ECRs, allowing equal engagement and direct interaction (by considering technology access and country representation)	Empower new collaborations in an inclusive manner, especially considering interactions between the global North/South and by fostering South/South interconnections	Research output (publications, briefings, etc.) metrics, funding acquisition, representation metrics per country/region	Promote regional and transnational funding programs to stimulate hands-on cooperation between scientists
Understanding impact of COVID-19 on ECRs	International societies, academies, and organisations: consider subsidising internet access to members and create small funding programs	Increase virtual engagement	Metrics on participation and demographics, assessment of newly created programs for ECR development	Fostering multinational investment allocation in projects directed by young researchers
Reduction of funding impacting professional networking activities	Conference, workshop, webinar, and event organizers: hold events when possible free of charge or with low/differential cost to science-lagging countries; open the event for all sectors (academia, industry, business, policy); learn from best practices	Support open science for all; increase sectors engagement; support science-policy and science-stakeholders discourse	Metrics on participation and demographics	Promote (a) scientific mobility to international and intraregional loci; and (b) scientific regular virtual meetings between international scientists and other stakeholders (this kind of meetings is a pandemic response/practice that likely will remain in the future)
Reduction in scientific funding	International societies, academies, and organisations: advocate with governments and institutions to present the importance of other fields of science despite the international emergency we are facing and aim to influence public budget decisions	Mitigate effect on future funding and influence decision-making on current funding calls.	Changes in budget allocation, incorporation of new actors in the funding landscape, such as NGOs and philanthropic groups	Engaging in regional legislations (a) to advocate for common minimum GDPs amounts for funding science and reduce regional scientific budgetary differences and gaps, and (b) to promote private investment in science allied with academia

The challenges identified in the first column, represent those originated or augmented by the COVID-19 pandemic.

*ODA* Official development assistance.

## Early-career researchers in global engagement, policy and diplomacy: an example with Young Academies

The Global Young Academy (GYA) is a worldwide organisation of early and mid-career scientists from different disciplines and countries. It empowers young researchers to lead international, interdisciplinary, and intergenerational dialogues to create an impact on science and society. GYA has served as a supportive institution promoting the foundation of novel National Young Academies (NYAs) worldwide. With the support and/or communication with the GYA, many NYAs have been established, like in Bangladesh, Hungary, and D.R. Congo. Interestingly, the new D. R. Congo Young Academy of Sciences (DRC-YAS) was supported by the GYA in conjunction with other established African NYAs^7^. Members of these academies have engaged in debates that served to modulate international engagement of their countries from a scientific realm (A 21st-Birthday Wish for Young Academies of Science, 2021; Bálint et al., 2021). NYAs have promoted science advice at the national level and science diplomacy and scientific collaboration at the regional level and worldwide, many times with GYA participation. For example, Young Academies Science Advice Structure (YASAS) was established in 2020 with the GYA being an executive board member^8^.

The World Health Organisation declared COVID-19 a pandemic on March 11, 2020. To support the pandemic response, on March 26, the GYA released the statement “Beyond Boundaries: A global message from young scientists on COVID-19”, with recommendations to strengthen international partnerships, as well as to formulate harmonised international scientific policies toward the mitigation of the pandemic effects (Global Young Academy, 2020). On the axis Science in Diplomacy, as part of the G7 group of academies, GYA is able to integrate the voices of ECRs with the advocacy of diplomatic groups; as seen with the release of the statement “The critical need for international cooperation during Covid-19 pandemic joint statement of Academies of Sciences and Medicine”^9^. Also, the G20 group of Science Academies (S20), released the statement “Foresight: Science for Navigating Critical Transitions”,^10^ with participation of GYA members. This statement provides recommendations on how we navigate the crisis and transition, as a global catastrophe can affect multiple aspects of our lives, such as economies, health and social security systems, and capacities to respond with robust scientific knowledge to guide national, regional, and global policies. This *communiqué* directly reported to policymakers and/or governments of G20 countries.

The initial months of the pandemic highlighted the need for inclusive science communication that helps inform general audiences about the need of science diplomacy and science advice to tackle solutions to the pandemic. The webinar “COVID-19 in Latin America, perspectives from young scientists” in May 2020, brought together six GYA scientists to provide attendees from 20 countries with reliable and accurate information on the pandemic^11^.

Starting in 2020, the accelerated transition to online interactions as a consequence of COVID restrictions, created more inclusive settings by eliminating travel-associated logistics and monetary factors as limitations. Often, travel expenses for in-person meetings of academies may not be allocated from research grants or fellowships as they are considered service, hindering participation. The GYA annual general meeting, e-AGM and e-conference were held fully online in the past two consecutive years (June 2020 and June 2021). The previous one combined real-time interactive and asynchronous events, and discussions with pre-recorded presentations, and the second one was fully live. Both years had some of the highest attendance rates, despite encompassing attendees challenged by the differences in time zones. However, the digital divide, which existed before the pandemic, has surfaced more than ever now that most events are virtual. One aspect is suboptimal or lack of suitable technology in some regions—a major challenge for synchronic engagement on global events with a diverse vision. A practical measure to enable equal opportunities for synchronous interactions is to subsidise internet access for participants in a specific event as fees might be unaffordable in some countries (Table 1)^12^. The mentioned e-events offered new, experimental ways of engagement for GYA members, representatives of NYAs, renowned scientists, professionals in science policy, along with key partner organisations^13^. This virtualisation of conferences due to the pandemic, was also observed for other GYA activities that fostered collaboration through presential experiences in another country. For example, in 2020, the bilateral Young Scientist Ambassador Program (YSAP)^14^ YSAP missions were modified to a virtual format. Time will determine if their impact is similar to the in-person format^15^, where there was a possibility to really experience research in another country and to realise direct outreach activities with the community during the visit.

Another aspect of the digital divide is the paywall to access scientific literature and publication fees, that some publishers may have tried to counteract with tuition or fee waivers, but which may still be aggravated by reduced funding. In fact, it might be worse during the pandemic in institutions with no remote access to their digital library during closures. Interestingly, most of COVID-19-related research has been made open access, even after being published in peer-reviewed journals, which normally have article access through payment. This measure has evidenced the urgency to advocate for equitable open access for all other disciplines, a trend that must be promoted and expanded. The GYA advocates for open science through partnering with UNESCO and other key stakeholders in a wide range of initiatives. This rapport includes workshops, consultations, and participation in high-level dialogues.

The GYA and NYAs work together to increase scientific collaborations in different regions and suggest solutions from ECRs to address globally important issues. With the new virtuality accelerated by the COVID-19 pandemic, the first online global meeting of NYAs, organised by GYA in September 2020, allowed the largest gathering of young academies so far (70 representatives from over 40 young academies), represented a global platform for establishing further collaborations and exchange of best practices between ECRs and different decision-making capabilities within academic, private, and governmental sectors. This meeting served to gather Latin American and Caribbean fellows involved in the writing of a current assessment on the situation of early- and mid-career scholars in this region that would include the long-term effect of pandemic on different topics like intraregional scientific collaboration and mobility, scientific practices between countries, and lack of resources for scientific research Such report and similar ones are planned to be distributed to various stakeholders in this region with different degrees of decision-making competences (Lopez-Verges et al., 2021; McAlpine et al., 2020; Miranda-Nieto et al., 2021), as it has been previously done for the ASEAN region (Geffers et al., 2017).

To suggest tailored solutions, further effects of the pandemic on ECRs must be assessed. Members of the GYA Women in Science working group compiled experiences amongst members, women scientists from different countries, as they tried to navigate their work and motherhood. They shared pieces of advice and motivation in the article “GYA Women in Science stay and work from home: How might we make COVID-19 lockdown work for us?”^16^. This exploratory perspective was applied to other projects, like the inclusion of COVID-19-related questions in different surveys, such as the impact of the pandemic in women in science’s work, or on ECRs from a specific region through the Global State of Young Scientists in Latin America and the Caribbean study (GLoSYS LAC). The National Young Academy of Nepal (NaYAN) recently conducted an analysis of the state of ECRs in Nepal and reported several barriers to conduct research in a low-resource setting^17^ Other young academies have been actively advocating for ECRs during the COVID-19 pandemic, assessing the issues ECRs are currently facing in their countries and discussing the future of research output after COVID-19 (Dékány et al., 2020) (examples in endnotes from the Young Academies of Cameroon, Israel, Hungary, The Netherlands and Young Academy of Europe)^18^.

Capacity building for global partnerships is crucial all the time, but even more necessary in these times that science needs collaborative young leaders with science communication, advice and diplomacy skills. The Science Leadership Programs (SLPs) implemented by GYA in Africa and Asia, are tools that could be adapted for current specific needs and be expanded to other regions. Additionally, a specific space to build science diplomacy skills of ECRs in South Asia was developed as a virtual workshop^19^.

## Learning from the described experiences to support early-career researchers and how they could help mitigate the effect of the pandemic on ECRs

A global perspective on issues emerging from the COVID-19 pandemic contingency and impact on ECRs have been presented in this commentary article. ECRs represent one of the most trained workforces as they continue advancing their careers. This pandemic has shown that science is needed to solve global challenges, and that ECRs, despite being the future of science, are highly impacted during a crisis of this magnitude, making their career progression very vulnerable. Thus, to continue developing and strengthening sustainable, innovative and impactful science for a globally sustainable and equitable development, there is a need to solve these challenges faced by ECRs through different strategies and actions, including science diplomacy and science policy approaches. The GYA and NYAs activities briefly introduced in this commentary, constitute experiences on how international associations can foster partnerships to act proactively and have an impact in ECRs life. Similarly, supporting the recommendations listed below might be useful and considered as intersectional issues that can be developed in future science diplomacy or science advice engagements. Based on this, general suggestions are proposed for scientists and policymakers to employ science advice and science diplomacy tools to address and overcome these urgent issues on ECRs. The monitoring, evaluation, and analysis of the impact of implementing these recommendations would be helpful to continuously improve the measures, as overcoming challenges faced by ECRs will strengthen the countries’ scientific systems. Thus, as science plays a crucial role in development, it would help create a sustainable society that could be resilient and prepared to face the new challenges of tomorrow.

## Footnotes

1 https://www.covidclinical.net/members/index.html

2 https://ourworldindata.org/covid-stay-home-restrictions

3 https://www.natureindex.com/news-blog/decline-women-scientist-research-publishing-production-coronavirus-pandemic. Also, an analysis by M. Frederickson, COVID-19’s gendered impact on academic productivity https://github.com/drfreder/pandemic-pub-bias/blob/master/README.md

4 Some of these programs have been developed through science diplomacy by the participating countries with the vision of “diplomacy for science” like the CYTED networks for the Iberoamerican region https://www.segib.org/programa/cyted-programa-iberoamericano-de-ciencia-y-tecnologia-para-el-desarrollo/

5 https://www.science.org.au/files/userfiles/support/documents/covid19-emcr-impact-report.pdf

6 https://www.hepi.ac.uk/2020/03/16/what-might-covid-19-mean-for-phd-students-postdocs/

7 https://globalyoungacademy.net/congo-young-academy-of-sciences-launched/

8 YASAS https://yacadeuro.org/press-release-fourteen-young-academies-in-europe-prepare-to-deliver-science-advice/

9 https://globalyoungacademy.net/gscience-gya-covid-19-statement/

10 G20 group of Science Academies S20 statement https://council.science/wp-content/uploads/2020/11/2020_S20SACommunique.pdf

11 https://globalyoungacademy.net/covid-19-in-latin-america-perspectives-from-young-scientists-webinar-report/

12 The GYA provided such a subsidy for connecting to the e-AGMs in cases where members required it.

13 https://globalyoungacademy.net/2020-emeetings-healtheearth/; GYA 2021 AGM and International Conference “Trust in Science” | Global Young Academy

14 https://globalyoungacademy.net/activities/young-scientist-ambassador-program/

15 As this is a recent program, anecdotal data support the contention that this endeavour set out networks that are currently, - and likely in the future - producing research outputs in the form of reports and peer-reviewed publications.

16 https://globalyoungacademy.net/wp-content/uploads/2020/05/GYA-WiS-Paper-May2020.pdf - This open-access booklet was introduced at the S20 Women in Science webinar (G20 Science Summit) in September 2020: https://globalyoungacademy.net/gya-members-help-shape-the-g20-science-20-summit/

17 The National Young Academy of Nepal survey on the state of ECRs in Nepal NaYAN - Download (youngacademynepal.org)

18 ECR advocacy during the pandemic, Cameroon Academy of Young Scientists (CAYS) https://globalyoungacademy.net/wp-content/uploads/2020/10/Report-of-CAYS-webinar-series-Sep2020.pdf; Israel Young Academy http://www.young.academy.ac.il/SystemFiles/ENG%202020-11-13%20IYA-COVID.pdf; Hungarian Young Academy https://globalyoungacademy.net/wp-content/uploads/2020/06/HYA-Covid-statement.pdf; The Young Academy of The Netherlands https://www.scienceguide.nl/2020/04/reward-and-appreciate-solidarity-in-times-of-crisis/; Young Academy of Europe http://yacadeuro.org/survey-on-the-impact-of-covid-19-pandemic-on-researchers/

19 The Science Diplomacy in South Asia workshop had participants from 7/8 South Asian countries. Remarkably, not only in this event but in the organising working group India and Pakistan are represented, stressing the need and willingness of science in/for diplomacy between countries with recent and current political tensions. https://globalyoungacademy.net/call-science-diplomacy-for-asia-workshop/
