# Correction: Mitigating losses: how scientific organisations can help address the impact of the COVID-19 pandemic on early-career researchers

**DOI:** 10.1057/s41599-021-01000-8

**Published:** 2021-12-02

**Authors:** Sandra López-Vergès, Bernardo Urbani, David Fernández Rivas, Sandeep Kaur-Ghumaan, Anna K. Coussens, Felix Moronta-Barrios, Suraj Bhattarai, Leila Niamir, Velia Siciliano, Andreea Molnar, Amanda Weltman, Meghnath Dhimal, Shalini S. Arya, Karen J. Cloete, Almas Taj Awan, Stefan Kohler, Chandra Shekhar Sharma, Clarissa Rios Rojas, Yoko Shimpuku, John Ganle, Maryam M. Matin, Justine G. Nzweundji, Abdeslam Badre, Paulina Carmona-Mora

**Affiliations:** 1 Gorgas Memorial Institute of Health Studies, Sistema Nacional de Investigación SNI del SENACYT, Panama City, Panama; 2 Center for Anthropology, Venezuelan Institute for Scientific Research, Caracas, Venezuela; 3 University of Twente, Enschede, The Netherlands; 4 Young Academy of Europe, Cardiff, UK; 5 University of Delhi, New Delhi, India; 6 Organization for Women in Science for the Developing World, Trieste, Italy; 7 Walter and Eliza Hall Institute of Medical Research, Parkville, VIC Australia; 8 University of Cape Town, Cape Town, South Africa; 9 International Centre for Genetic Engineering and Biotechnology, Trieste, Italy; 10 Global Institute for Interdisciplinary Studies, Lalitpur, Nepal; 11 National Young Academy of Nepal, Kathmandu, Nepal; 12 International Institute for Applied Systems Analysis (IIASA), Laxenburg, Austria; 13 Mercator Research Institute on Global Commons and Climate Change, Berlin, Germany; 14 Istituto Italiano di Tecnologia, Naples, Italy; 15 Swinburne University of Technology, Hawthorn, VIC Australia; 16 Early- and Mid-Career Researcher Forum, Melbourne, VIC Australia; 17 Nepal Health Research Council, Kathmandu, Nepal; 18 Institute of Chemical Technology, Mumbai, India; 19 Indian National Young Academy of Sciences, New Delhi, India; 20 UNESCO-UNISA Africa Chair in Nanosciences/Nanotechnology, College of Graduate Studies, University of South Africa, Pretoria, South Africa; 21 Nanosciences African Network (NANOAFNET), iThemba LABS-National Research Foundation, Somerset West, South Africa; 22 University of Sao Paulo, Sao Paulo, Brazil; 23 National Young Academy of Young Scientists Pakistan, Lahore, Pakistan; 24 Heidelberg University, Heidelberg, Germany; 25 Indian Institute of Technology, Hyderabad, India; 26 Centre for the Study of Existential Risk, University of Cambridge, Cambridge, UK; 27 Hiroshima University, Hiroshima, Japan; 28 Young Academy of Japan, Tokyo, Japan; 29 University of Ghana, Accra, Ghana; 30 Department of Biology, Ferdowsi University of Mashhad, Mashhad, Iran; 31 Institute of Medical Research and Medicinal Plants Studies, Yaounde, Cameroon; 32 Cameroon Academy of Young Scientists, Yaounde, Cameroon; 33 Mohammed V University in Rabat, Rabat, Morocco; 34 Department of Neurology and MIND Institute, University of California-Davis, Sacramento, CA USA

**Keywords:** Science, technology and society, Health humanities

Correction to: *Humanities and Social Sciences Communications* 10.1057/s41599-021-00944-1, published online 19 November 2021.

A correction has been made to the online article and PDF to ensure one of the author’s affiliations was correctly listed.

The original text stated that Felix Moronta-Barrios was affiliated with:

- International Centre for Genetic Engineering and Biotechnology, Trieste, Italy.
- International Institute for Applied Systems Analysis (IIASA), Laxenburg, Austria.

This has now been corrected, so that Felix Moronta-Barrios is only affiliated with:

- International Centre for Genetic Engineering and Biotechnology, Trieste, Italy.

